# Apolipoprotein E levels in cerebrospinal fluid and the effects of *ABCA1 *polymorphisms

**DOI:** 10.1186/1750-1326-2-7

**Published:** 2007-04-12

**Authors:** Suzanne E Wahrle, Aarti R Shah, Anne M Fagan, Scott Smemo, John SK Kauwe, Andrew Grupe, Anthony Hinrichs, Kevin Mayo, Hong Jiang, Leon J Thal, Alison M Goate, David M Holtzman

**Affiliations:** 1 Department of Neurology, Washington University, St. Louis, MO, USA; 2 Department of Psychiatry, Washington University, St. Louis, MO, USA; 3 Celera Diagnostics, Alameda, CA, USA; 4 Department of Medicine, Division of Biostatistics, Washington University, St. Louis, MO, USA; 5 Department of Neurosciences, University of California at San Diego, San Diego, CA, USA; 6 Department of Genetics, Washington University, St. Louis, MO, USA; 7 Department of Molecular Biology and Pharmacology, Washington University, St. Louis, MO, USA

## Abstract

**Background:**

Animal studies suggest that brain apolipoprotein E (apoE) levels influence amyloid-β (Aβ) deposition and thus risk for Alzheimer's disease (AD). We have previously demonstrated that deletion of the ATP-binding cassette A1 transporter (ABCA1) in mice causes dramatic reductions in brain and cerebrospinal fluid (CSF) apoE levels and lipidation. To examine whether polymorphisms in *ABCA1 *affect CSF apoE levels in humans, we measured apoE in CSF taken from 168 subjects who were 43 to 91 years old and were either cognitively normal or who had mild AD. We then genotyped the subjects for ten previously identified *ABCA1 *single nucleotide polymorphisms (SNPs).

**Results:**

In all subjects, the mean CSF apoE level was 9.09 μg/ml with a standard deviation of 2.70 μg/ml. Levels of apoE in CSF samples taken from the same individual two weeks apart were strongly correlated (r^2 ^= 0.93, p < 0.01). In contrast, CSF apoE levels in different individuals varied widely (coefficient of variation = 46%). CSF apoE levels did not vary according to AD status, *APOE *genotype, gender or race. Average apoE levels increased with age by ~0.5 μg/ml per 10 years (r^2 ^= 0.05, p = 0.003). We found no significant associations between CSF apoE levels and the ten *ABCA1 *SNPs we genotyped. Moreover, in a separate sample of 1225 AD cases and 1431 controls, we found no association between the *ABCA1 *SNP rs2230806 and AD as has been previously reported.

**Conclusion:**

We found that CSF apoE levels vary widely between individuals, but are stable within individuals over a two-week interval. AD status, *APOE *genotype, gender and race do not affect CSF apoE levels, but average CSF apoE levels increase with age. Given the lack of association between CSF apoE levels and genotypes for the *ABCA1 *SNPs we examined, either these SNPs do not affect ABCA1 function or if they do, they do not have strong effects in the CNS. Finally, we find no evidence for an association between the *ABCA1 *SNP rs2230806 and AD in a large sample set.

## Background

Alzheimer's disease (AD) is an age-related progressive neurodegenerative disorder that causes impairments in memory and thinking. The strongest genetic risk factor for AD is apolipoprotein E (*APOE*) genotype [1]. In comparison to people who are homozygous for the common ε3 allele, people who carry the ε4 allele are at higher risk for AD and generally have an earlier age of onset, while people who carry the ε2 allele are at lower risk and have a later age of onset [2-6]. ApoE is a chaperone for amyloid-β (Aβ) peptide, which deposits in the brain and is thought to initiate a cascade of events that causes AD [7,8]. Mouse models have shown that the time of onset and amount of Aβ deposition depends not only on *APOE *genotype but also on apoE levels. Interestingly, higher expression of mouse apoE increases the amount of Aβ deposition [9,10], while higher expression of the human ε3 isoform of *APOE *knocked into the mouse *Apoe *locus decreases levels of amyloid deposition [11]. Additionally, expression of human apoE in mice delays the onset of Aβ deposition in an isoform-specific fashion, with ε2 expression decreasing Aβ deposition the most and ε4 expression decreasing Aβ deposition the least [12,13].

Despite evidence from animal studies suggesting that apoE levels affect Aβ deposition, there is no consensus regarding levels of apoE expression and its effects on Aβ deposition in human studies. The examination of whether apoE levels affect AD risk in humans has focused on *APOE *promoter polymorphisms. Over 50 studies listed on the Alzforum website tested for an association between AD and one or more polymorphisms within the *APOE *promoter [14]. Meta-analyses on this website support the notion that *APOE *promoter variation is associated with risk for AD. However, it is unclear whether this association is due to linkage disequilibrium with the coding polymorphisms or whether there are independent effects on risk due to the level of *APOE *expression. Some studies have examined the effect of *APOE *promoter polymorphisms on *APOE *expression *in vitro *[15,16]. More recently, allele specific gene expression has been used in post-mortem brain samples to measure the relative expression of *APOE *ε3 and ε4 isoforms [17]. However, even these studies do not directly examine the effect of the promoter polymorphisms on levels of apoE protein.

Previous studies of CSF apoE levels in humans have reached varying conclusions. Some report that CSF apoE levels are lower in AD subjects than in control subjects [18-20], other studies find no association between CSF apoE levels and AD [21,22], and one study shows that CSF apoE levels are higher in AD subjects than in control subjects [23]. Multiple studies found that the *APOE *genotype was not associated with differing CSF apoE levels [19-22]. In contrast, plasma apoE levels are clearly dependent on *APOE *genotype [24,25], which suggests that apoE is metabolized differently in the CSF and plasma. Gender and age do not appear to affect CSF apoE levels [22].

Recently, our laboratory and others reported that apoE levels were greatly reduced in mice lacking functional ATP-binding cassette A1 transporter (ABCA1) [26-28]. Within the CNS of ABCA1 knock-out mice, CSF apoE was 2% of normal levels and apoE in the cortex was 20% of normal levels [26]. ABCA1 transfers cholesterol and phospholipids from the cell membrane to apolipoproteins (including apoE) to form nascent high density lipoproteins (HDL). In the rare case that both copies of *ABCA1 *are non-functional, as occurs in Tangier's disease, apoE and other lipoproteins do not receive normal amounts of lipid and are rapidly degraded [29]. Multiple studies have shown that levels of plasma HDL-C and associated apolipoproteins are affected by single nucleotide polymorphisms (SNPs) in *ABCA1 *[30-34]. In particular, studies have implicated the following SNPs in affecting levels of plasma HDL-C: rs2230806 (R219K) [33], rs2066718 (V771M) [31,32], rs2066715 (V825I) [31], rs4149313 (I883M) [34], rs2230808 (R1587K) [31]. Since ABCA1 appears to have a similar role in the CNS and in the periphery, we hypothesized that these *ABCA1 *SNPs would also have an effect on CSF apoE levels since apoE is the major apoprotein component of HDL produced in the CNS. Additionally, studies by others have reported that the *ABCA1 *SNP rs2230806 (R219K) affects risk for AD [35-38]. This is particularly interesting because *ABCA1 *falls within a region of chromosome 9 that is linked to late-onset AD [39-43]. The profound effect of ABCA1 levels on CNS apoE levels in mice, in addition to reports that an *ABCA1 *SNP may affect risk for AD, suggested that *ABCA1 *may be involved in the genetic control of CNS apoE levels in humans.

Given the contrasting results and small sample sizes used in some studies of apoE levels in human CSF, we chose to begin our study by characterizing CSF apoE levels in a relatively large sample of 168 individuals with respect to AD status, *APOE *genotype, gender, race and age. We next examined whether ten *ABCA1 *SNPs, including five SNPs shown to affect plasma HDL-C, affected levels of apoE in the CSF. Finally, in a large sample of 1225 AD cases and 1431 controls, we attempted to replicate the previously reported association between the *ABCA1 *SNP rs2230806 and AD.

## Results

### ApoE levels and stability in human CSF

**Figure 1:**
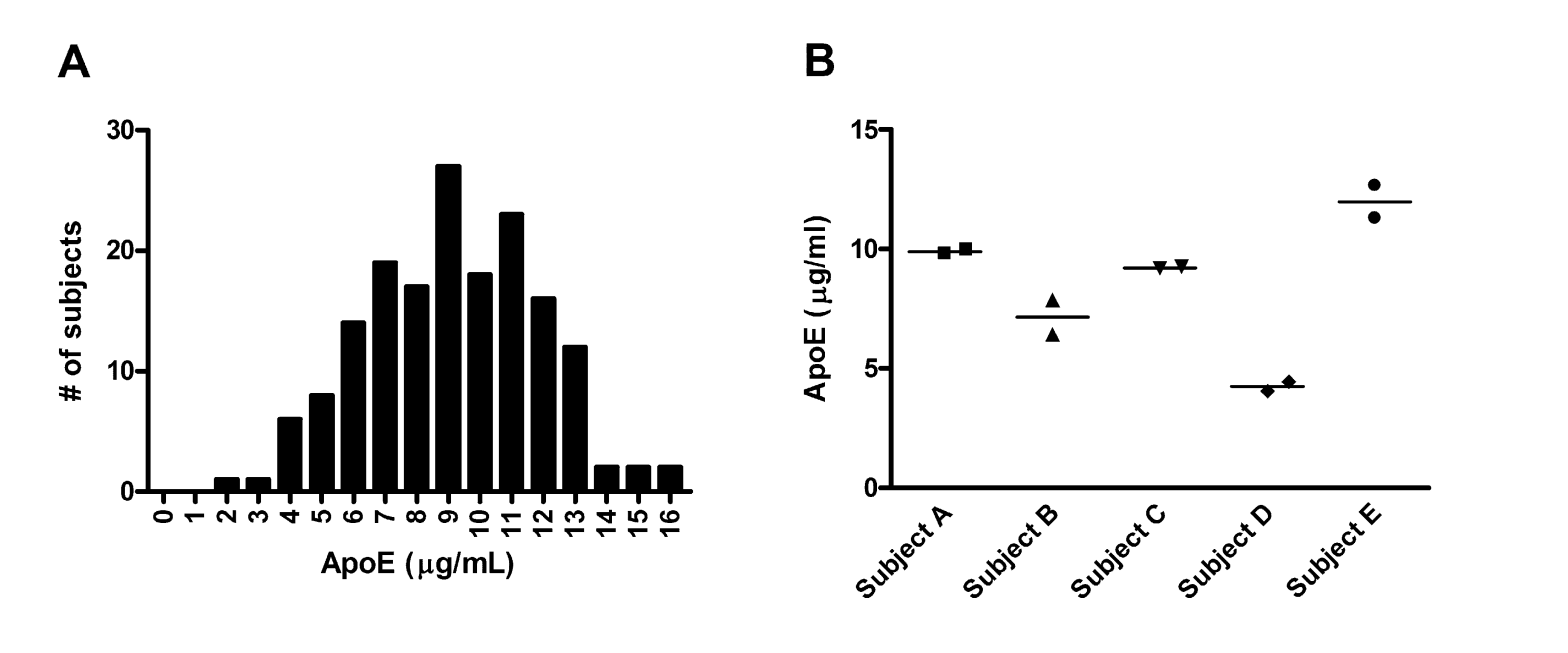
Distribution of apoE levels in human CSF. A, ApoE levels were sorted into bins of 1 μg/ml and the number of subjects with apoE values within each bin was tallied. The data represents 168 subjects without division by CDR status, APOE genotype, gender, race or age. B, ApoE levels were measured in CSF samples taken two weeks apart from five different patients.

ApoE levels were measured in CSF samples from 168 subjects who were 43 to 91 years old (Table 1). We included all samples available without regard to AD status, *APOE *genotype, gender, race or age. ApoE values were sorted into 1 μg/ml bins and the number of subjects with apoE values within each bin from 0 to 16 μg/ml was tallied (Fig. 1A). The mean apoE level was 9.09 μg/ml with a standard deviation of 2.70 μg/ml. The number of individuals per bin was in a normal distribution according to a Kolmogorov-Smirnov test (p > 0.10).

**Table 1:** Characteristics of subjects who underwent lumbar puncture.

	**CDR 0, <65**	**CDR 0, ≥65**	**CDR 0.5**	**CDR 1+**
**n =**	70	55	26	17
**Male**	29%	28%	54%	47%
**Female**	71%	72%	46%	53%
Age*	54 ± 6	76 ± 8	75 ± 8	76 ± 6
**ε2 freq.**	0.11	0.13	0.06	0.03
**ε3 freq.**	0.64	0.73	0.56	0.74
**ε4 freq.**	0.25	0.14	0.38	0.24

*Age is mean ± standard deviation

To determine the intra-individual stability of CSF apoE levels sampled over time, lumbar puncture was performed on five subjects at two different times that were two weeks apart. CSF apoE levels within the same individual were strongly correlated (r^2 ^= 0.93, p < 0.01). In contrast, CSF apoE levels between different individuals showed large variation (coefficient of variation = 46%) (Fig. 1B). This demonstrates that CSF apoE levels are relatively stable within an individual during a short time interval, but vary widely between individuals. Furthermore, this suggests that CSF apoE levels may be influenced by stable individual differences, such as genetic sequence variation.

### Effects of AD status, APOE genotype, gender or age on CSF apoE levels

**Figure 2:**
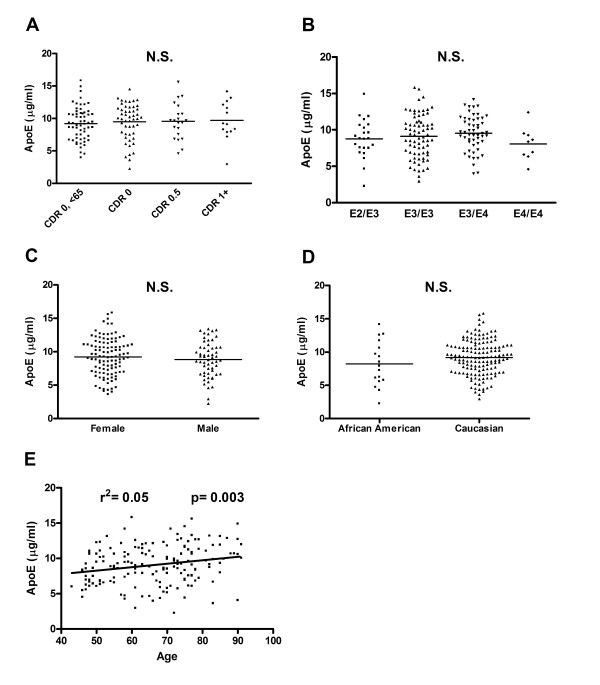
ApoE levels in human CSF do not vary according to presence or absence of Alzheimer's disease, level of cognitive impairment, *APOE *genotype, gender or race, but do increase with age. A, Subjects were grouped by age and AD status. Subjects with a clinical dementia rating (CDR) score of 0 (cognitively normal) that were less than age 65 were placed into the first group (CDR 0, <65; n = 59). Subjects that were 65 and older with a CDR score of 0, 0.5, or 1–2 were placed into the second (CDR 0, n = 50), third (CDR 0.5, n = 21) and fourth (CDR 1+, n = 14) groups, respectively. There was no difference in CSF apoE levels by one-way ANOVA. B, Subjects were grouped by *APOE *genotype into four groups: E2/E3 (n = 23), E3/E3 (n = 72), E3/E4 (n = 52), and E4/E4 (n = 9). There was no difference in CSF apoE levels by one-way ANOVA. C, Subjects were divided into two groups, female (n = 109) and male (n = 57). There was no difference in CSF apoE levels by a two-tailed Student's T-test. D, Subjects were grouped by self-identified racial group: African American (n = 17) and Caucasian (n = 149). There was no difference in CSF apoE levels by a two-tailed Student's T-test. E, CSF apoE levels were graphed as a function of subject age (n = 168). The slope of the regression line was 0.05, with a 95% confidence interval of 0.02 to 0.08.

There are varying reports in the literature on whether CSF apoE levels are affected by AD status, *APOE *genotype, gender or age. In our relatively large sample, we investigated whether these variables, as well as race, modified CSF apoE levels. The levels of CSF apoE were not significantly different between subjects who were cognitively normal who had a clinical dementia rating (CDR) score of 0 and those who had very mild (CDR 0.5) or mild-moderate dementia believed to be due to AD (CDR 1+) (Fig. 2A). Since a recent study reported that apoE levels may be affected by *APOE *genotype [44], we examined whether *APOE *genotype affects CSF apoE levels in our sample. Despite large numbers of patients, we found no significant differences in CSF apoE levels in subjects with different *APOE *genotypes (Fig. 2B). Next, we looked for gender effects on CSF apoE levels and found none (Fig. 2C). We also found no significant difference in CSF apoE levels between subjects who identified themselves as Caucasians and African Americans (Fig. 2D). Additionally, we studied whether age affects CSF apoE levels (Fig. 2E). Average apoE levels increased by a small but significant extent, ~0.5 μg per 10 years (r^2 ^= 0.05, p = 0.003). Finally, to test the possibility that AD status, *APOE *genotype, gender and age interact to influence apoE levels in the CSF, we performed a multivariate ANOVA and found no significant interactions. We conclude that CSF apoE levels are not greatly affected by AD status, *APOE *genotype, gender or race, but do increase with age.

### Effects of ABCA1 SNPs on CSF apoE levels and risk for AD

We sought to determine whether SNPs in *ABCA1 *affect CSF apoE levels. The subjects for whom we had CSF apoE data were genotyped for the following *ABCA1 *SNPs: rs2230806 (R219K), rs2066718 (V771M), rs2066715 (V825I), rs4149313 (I883M), rs2230808 (R1587K), rs1883025 (intron), rs2275544 (intron), rs2777799 (intron), rs3904999 (intron) and rs6479283 (intron). The numbers of subjects for which we obtained conservatively called (high quality) genotypes, as well as the frequencies of the minor and major alleles, are listed in Table 2. We found no association between CSF apoE levels and any of the *ABCA1 *SNPs, including the five coding SNPs that were previously associated with alterations in plasma HDL-C levels.

**Table 2:** The number of subjects with high quality genotypes and the frequency of the minor and major ABCA1 SNP alleles.

	**n =**	**minor allele freq.**	**major allele freq.**
**rs2230806**	123	0.309	0.691
**rs2066718**	124	0.040	0.960
**rs2066715**	144	0.073	0.927
**rs4149313**	124	0.185	0.815
**rs2230808**	124	0.315	0.685
**rs1883025**	102	0.358	0.642
**rs2275544**	122	0.131	0.869
**rs2777799**	123	0.126	0.874
**rs3904999**	123	0.203	0.797
**rs6479283**	119	0.223	0.777

We also attempted to reproduce the finding, reported by some groups but not others, that the *ABCA1 *rs2230806 SNP is associated with altered risk for AD [35-38,45]. We combined information on 794 subjects from Washington University with 1,862 additional subjects from the University of California-San Diego and the United Kingdom to yield the maximum power. The subjects from Washington University had previously been analyzed and it was found that risk for AD in this group did not depend on the rs2230806 SNP [36]. The 1,862 additional subjects had not previously been used to examine the rs2230806 SNP. In this large group of 1225 case and 1431 control subjects, there was no effect of the rs2230806 SNP on risk for AD (Table 3). Analysis of sub-groups based on *APOE *genotype and gender also failed to show an effect of the rs2230806 SNP on risk for AD.

**Table 3:** The distribution of the rs2230806 polymorphism in subjects with Alzheimer's disease and control subjects.

		*#*	# AA	# AG	*# *GG	freq. A	freq. G	AD vs. Control
**Total**	AD	1225	81	476	668	0.260	0.740	**p = 0.76**
n = 2656	Control	1431	105	548	778	0.265	0.735	
**E3/E3**	AD	437	31	170	236	0.265	0.735	p = 0.93
n = 1316	Control	879	63	351	465	0.271	0.729	
**E4/E3**	AD	555	32	227	296	0.262	0.738	p = 0.10
n = 832	Control	277	18	92	167	0.231	0.769	
**E4/E4**	AD	125	8	40	77	0.224	0.776	p = 0.86
n = 150	Control	25	1	9	15	0.220	0.780	
**Females**	AD	267	26	105	136	0.294	0.706	p = 0.99
n = 505	Control	238	23	94	121	0.294	0.706	

p values are caculated by Chi Square tests with 2 degrees of freedom

## Discussion

A notable finding in this study was that CSF apoE levels vary widely between individuals, with a range in our sample from 2 μg/ml to 16 μg/ml, but are stable within individuals during an interval of 2 weeks. This suggests the presence of stable factors within individuals, which may be genetic or environmental, that regulate CSF apoE levels. Recently, it was reported that levels of Aβ vary according the time of day and it is possible that apoE could vary in a similar fashion [46]. However, since all of our samples were obtained at the same time of day (8:00 am), any diurnal variation of apoE levels in this study should be minimal.

We examined whether AD status, *APOE *genotype, gender, race or age affected CSF apoE levels, but only age was significantly correlated. It is interesting that levels of apoE are not elevated in carriers of the ε2 allele. ApoE3 and apoE4 both bind with high affinity to LDLR resulting in receptor-mediated endocytosis and degradation of apoE. ApoE2 does bind to LDLR, but much less effectively than apoE3 and apoE4 [47]. In mice, the decreased affinity of apoE2 for LDLR leads to elevated levels of CSF apoE in mice in which the human *APOE *ε2 gene is knocked-in to the mouse *Apoe *gene locus [48]. The lack of a difference in apoE levels according to genotype in human CSF samples suggests that LDLR may not have as large of an effect on human CSF apoE levels. It will be important to assess this issue in future studies in *APOE *ε2 homozygous individuals as there may be a much smaller effect in individuals with one copy of the *APOE *ε2 gene.

We hypothesized that genetic variation in certain genes may contribute to CSF apoE levels and examined whether SNPs in *ABCA1*, especially SNPs that have been reported to affect plasma HDL-C levels, affect CSF apoE levels. We did not find a significant association between CSF apoE levels and any of the ten *ABCA1 *SNPs we examined, including the five coding SNPs thought to be associated with altered HDL-C levels. Perhaps this is because the metabolism of apoE is different in the plasma and CSF. Alternatively, these changes in ABCA1 may not affect HDL in the CNS as much as occurs as with HDL in the plasma. This may be due to apoAI being the main apoprotein in plasma HDL whereas apoE is the most abundant apoprotein produced in the CNS in CSF HDL. The effects of the SNPs may also be too small to significantly affect CSF apoE levels. However, it remains possible that rare sequence variations that strongly influence *ABCA1 *function could contribute to variation in CSF apoE levels. Recent studies demonstrate that several rare polymorphisms in *ABCA1 *collectively affect overall levels of plasma HDL-C in the population [30,31]. Since ABCA1-mediated lipid transport is critical in the formation of both HDL-C in plasma and apoE-containing lipoproteins in CSF, it is possible that the same rare *ABCA1 *polymorphisms that have large effects on plasma HDL-C levels would also affect CSF apoE levels.

Additionally, we failed to replicate the finding of other groups that the *ABCA1 *rs2230806 SNP is associated with altered risk for AD [35-38]. We suggest three possible reasons for the differing results: 1) the *ABCA1 *rs2230806 SNP does affect risk for AD, but the effect is small so that the association cannot be reproduced regularly in samples of ~2500 subjects; or 2) the population we examined was genetically different from the populations in the other studies assessed; or 3) the *ABCA1 *rs2230806 SNP does not affect risk for AD. Since the populations that we and others examined are similar and consisted primarily of Caucasians with Northern European heritage, we believe that it is most likely that the *ABCA1 *rs2230806 SNP contributes either a very small amount or not at all to overall risk for AD.

It seems likely that many different genes modulate levels of apoE in the CSF. Studies suggest that LDLR and LRP influence levels of CSF apoE in mice [48,49]. Given the animal data, it is possible that variations in *LDLR *or *LRP *could affect CSF apoE levels in humans, but this has not yet been examined. Further investigation of the genetic control of apoE levels in the CNS could uncover new information on apoE metabolism. This research would not only be relevant to AD, but also to a number of other neurological diseases that may be modulated by apoE such as stroke [50,51], multiple sclerosis [52] and traumatic brain injury [53]. Ultimately, an understanding of the regulation of CSF apoE levels could lead to novel apoE-based treatments for AD and other neurological disorders.

## Conclusion

We found that CSF apoE levels vary widely between individuals, but are stable within individuals over a two-week interval. Secondly, AD status, *APOE *genotype, gender and race do not affect CSF apoE levels, but CSF apoE levels do increase with age. Additionally, ABCA1 SNPs that have been reported to affect plasma HDL-C levels do not affect CSF apoE levels in our sample. Finally, any association that exists between the *ABCA1 *SNP rs2230806 and AD is very weak.

## Methods

### Subjects

Subjects in the Washington University sample were community-living participants in the Alzheimer's Disease Research Center (ADRC) registry. All research subjects underwent a clinical evaluation to determine their Clinical Dementia Rating (CDR), as well as a 2-hour psychometric test battery. A medical history was taken to exclude participants that might have confounding medical disorders. Details of the assessment have been described previously [54-56]. Additional case control DNA samples were from the University of California-San Diego and the United Kingdom.

CSF was obtained via lumbar puncture (L.P.) from 168 subjects at Washington University in the General Clinical Research Center after obtaining informed consent. The study protocol was approved by the Human Studies Committee at Washington University. All L.P.s were performed at 8 am after an overnight fast with a 22 gauge atraumatic needle. 25–30 ml of CSF was obtained from each subject and was free of blood contamination. After collection, CSF samples were briefly centrifuged at 1,000 × *g *to pellet any cell debris, frozen, and stored in polypropylene tubes at -80°C in 0.5 ml aliquots until analysis.

### ApoE ELISA

ApoE ELISAs were performed on CSF apoE as previously described [48]. Briefly, plates were coated overnight with WUE4, a monoclonal antibody to human apoE [57]. The plates were washed, blocked with 1% dry milk and washed again. ApoE standards were purified from human β-VLDL (BioDesign, Sako, ME). Standards and samples were diluted and loaded onto the plate, then incubated overnight. The plate was washed and incubated with a polyclonal goat anti-apoE antibody (Calbiochem, San Diego CA). The plate was washed again and incubated with anti-goat-HRP (Vector Laboratories, Burlingame, CA). The plate was washed once more, then developed with TMB (Sigma, St. Louis, MO).

### Genotyping

The following SNPS in *ABCA1 *were genotyped in the Washington University sample of 168 subjects: rs2230806 (R219K), rs2066718 (V771M), rs2066715 (V825I), rs4149313 (I883M), rs2230808 (R1587K), rs1883025 (intron), rs2275544 (intron), rs2777799 (intron), rs3904999 (intron) and rs6479283 (intron). Genotyping was performed using a modified single nucleotide extension reaction with allele detection by mass spectrometry (Sequenom MassArray system; Sequenom, San Diego, CA, USA). PCR primers, termination mixes and multiplexing capabilities were determined with Sequenom Spectro Designer software v2.00.17. Genotyping of rs2230806 in the large group of 2,656 subjects was performed using allele specific real-time PCR [58]. For all SNPs, genotypes were tested and found to be in Hardy-Weinberg equilibrium.

### Statistical analyses

Frequency distributions, correlation analysis, ANOVAs, T-tests and Kolmogorov Smirnov tests of normality were performed using GraphPad Prism, Version 4.00 (GraphPad, San Diego, CA). Multivariate ANOVAs were performed using SAS Version 9.0 for Windows XP (SAS Institute Inc., Cary, NC).

## Abbreviations

Aβ, amyloid-β peptide; ABCA1, ATP-binding cassette transporter A1; AD, Alzheimer's disease; apoE, apolipoprotein E; CDR, clinical dementia rating; CNS, central nervous system; CSF, cerebrospinal fluid; ELISA, enzyme-linked immunosorbent assay; HDL, high density lipoprotein; LDLR, low density lipoprotein receptor; LP, lumbar puncture; LRP, low density lipoprotein related protein; SNP, single nucleotide polymorphism.

## Competing interests

The author(s) declare that they have no competing interests.

## Authors' contributions

SEW performed the primary writing and editing of the manuscript and was involved in experimental design, genotyping and data analysis. ARS processed CSF samples and assayed them for levels of apoE. AMF was involved in coordinating CSF collection and experimental design. SS, AG, KM, and HJ were involved in genotyping and experimental design.

JSKK and AH were involved in experimental design and statistical analysis. LJT provided samples from the UCSD collection. AMG and DMH were involved in experimental design, data analysis, and manuscript writing. All authors approved the manuscript.
